# Primary brain tumours following breast cancer

**DOI:** 10.1186/1753-6561-9-S1-A62

**Published:** 2015-01-14

**Authors:** M Hussain, JC Bolger, P O’Halloran, S McNally

**Affiliations:** 1 Royal College of Surgeons in Ireland, 123 St. Stephen’s Green, Dublin 2. Ireland; 2 Department of Surgery, Beaumont Hospital, Dublin 9, Ireland; 3 Department of Neurosurgery, Beaumont Hospital, Dublin 9, Ireland

## Background

Primary brain tumours account for one of the top ten reasons for all cancer-related death. It has previously been shown that there is an increased risk of developing a primary brain tumour following a prior solid tumour in the case of bladder cancer, endometrial cancer, sarcoma and leukaemia. There is no data on whether there is an increased risk in developing primary CNS neoplasia following breast cancer.

## Methods

Patient data was collected on all primary brain tumours diagnosed at Beaumont hospital between the years 2001-2013. This list of primary brain tumours was then cross-referenced with a set database of 4157 breast cancer patients. The result was then compared to the number we would expect in the average population over the same time period in a similar cohort that didn’t have breast cancer.

## Results

We calculated that we would expect 6.48 patients in a cohort of 4157 of the average population of women aged 40-74 between the years 2001-2013. 7 patients in our cohort of 4157 breast cancer patients developed a subsequent primary brain tumour. Thus there isn\'t a significant increase (relative risk 1.33, 95% confidence interval 0.46-3.83, p= 0.87) in the risk of acquiring a primary brain tumour in a patient that has had primary breast cancer.

## Conclusions

There is no statistically significant increase in risk of developing a primary brain tumour following breast cancer. This is new information that hasn\'t been reported before.

